# Applications of Machine Learning in Chronic Myeloid Leukemia

**DOI:** 10.3390/diagnostics13071330

**Published:** 2023-04-03

**Authors:** Mohamed Elhadary, Ahmed Adel Elsabagh, Khaled Ferih, Basel Elsayed, Amgad M. Elshoeibi, Rasha Kaddoura, Susanna Akiki, Khalid Ahmed, Mohamed Yassin

**Affiliations:** 1 College of Medicine, QU Health, Qatar University, Doha 2713, Qatar; 2 Pharmacy Department, Heart Hospital, Hamad Medical Corporation (HMC), Doha 3050, Qatar; 3 Diagnostic Genomic Division, Hamad Medical Corporation (HMC), Doha 3050, Qatar; 4 Department of Hematology, National Center for Cancer Care and Research (NCCCR), Hamad Medical Corporation (HMC), Doha 3050, Qatar; 5 Hematology Section, Medical Oncology, National Center for Cancer Care and Research (NCCCR), Hamad Medical Corporation (HMC), Doha 3050, Qatar

**Keywords:** artificial intelligence, chronic myeloid leukemia, machine learning, convolutional neural networks, hemoglobinopathies

## Abstract

Chronic myeloid leukemia (CML) is a myeloproliferative neoplasm characterized by dysregulated growth and the proliferation of myeloid cells in the bone marrow caused by the BCR-ABL1 fusion gene. Clinically, CML demonstrates an increased production of mature and maturing granulocytes, mainly neutrophils. When a patient is suspected to have CML, peripheral blood smears and bone marrow biopsies may be manually examined by a hematologist. However, confirmatory testing for the BCR-ABL1 gene is still needed to confirm the diagnosis. Despite tyrosine kinase inhibitors (TKIs) being the mainstay of treatment for patients with CML, different agents should be used in different patients given their stage of disease and comorbidities. Moreover, some patients do not respond well to certain agents and some need more aggressive courses of therapy. Given the innovations and development that machine learning (ML) and artificial intelligence (AI) have undergone over the years, multiple models and algorithms have been put forward to help in the assessment and treatment of CML. In this review, we summarize the recent studies utilizing ML algorithms in patients with CML. The search was conducted on the PubMed/Medline and Embase databases and yielded 66 full-text articles and abstracts, out of which 11 studies were included after screening against the inclusion criteria. The studies included show potential for the clinical implementation of ML models in the diagnosis, risk assessment, and treatment processes of patients with CML.

## 1. Introduction

Chronic myeloid leukemia (CML, also known as chronic myelogenous leukemia) is a clonal myeloproliferative neoplasm that accounts for approximately 15% of adult leukemias [1,2]. CML is characterized by the dysregulated proliferation of mature granulocytes (neutrophils, basophils, eosinophils) and their precursors. Notably, it was the first disorder to be linked to a particular chromosomal abnormality (i.e., Philadelphia (Ph) chromosome). Ph chromosome was later found to be formed due to a reciprocal translocation between chromosomes 9 and 22 that led to the fusion of the BCR gene (Chr. 22) and ABL1 gene (Chr. 9), forming the pathognomonic BCR-ABL fusion gene [3,4,5,6,7]. Clinically, CML can be divided based on progression into three phases: the chronic phase is when most patients are diagnosed; the accelerated phase characterized by impairment of neutrophil differentiation; and the blast phase in which myeloid cells proliferate uncontrollably, resembling acute leukemia [5,6,8,9]. The diagnosis of CML involves several steps, starting with a peripheral blood smear that typically shows neutrophilia, followed by bone marrow biopsy, and finally, testing for the BCR-ABL fusion gene or mRNA that are confirmatory for the disease [10,11,12,13,14].

Since the development of electronic records and the improvement in access and the retrieval of patient data (e.g., diagnostic tests, imaging, laboratory tests), terms such as ‘big data’ and ‘machine learning (ML)’ have started to have a meaningful impact on patient care in different aspects. ML refers to models or function approximators that learn to make conclusions or decisions based on information derived from raw data [15,16]. Currently, there are plenty of implementations of different ML models in the field of health care such as interpreting lab results, suggesting a favorable diagnosis based on imaging studies [17,18,19], giving treatment recommendations and classifying stages of disease. This has made ML increasingly important for many clinical scenarios in the modern health care system [20,21,22]. Therefore, ML models can be employed in the case of CML to aid in the workup process by predicting diagnosis or by improving patient management through predicting prognosis and giving treatment recommendations.

In this review, we summarize the recent literature pertaining to the use of ML algorithms in CML diagnosis, prognosis, and treatment. Furthermore, the review highlights the performance, limitations, and future research needs for the models reported. The goal of this review is to provide an overview and to fuel research on AI implementations in CML as it is well-embedded in clinical practice, and the use of ML algorithms could potentially enhance patient management and prognosis.

## 2. Materials and Methods

A comprehensive literature search strategy of all studies pertaining to ML implementations in CML diagnosis, prognosis, and treatment was conducted using the PubMed/MEDLINE and EMBASE databases. The search strategy used terms pertaining to CML (e.g., “chronic myeloid leukemia”, “chronic myelogenous leukemia”, “CML”, “CML-CP”) as well as terms of artificial intelligence (e.g., “AI”, “machine learning”, “ML”). After applying the search strategy, all of the identified studies were transferred to EndNote, where duplicates were eliminated. The resulting studies were then transferred to Rayyan to conduct further screening and remove any additional duplicates.

The primary literature that discussed the use of ML algorithms in different CML applications including full-text articles and conference abstracts were considered for inclusion in this review. The publication period from January 2012 until November 2022 was only considered. Articles that were excluded from this review were non-English articles, animal studies, in vitro studies, and review articles.

The collected data comprised several aspects including the type of study, publication year, assessed outcome, model creation methods, utilized model(s), and evaluation metrics for the model(s) such as sensitivity (SEN), specificity (SPE), accuracy (ACC), and area under the receiver operating curve (AUC). Additionally, the AUC values were categorized into different levels such as unsatisfactory (<0.6), satisfactory (0.6 to <0.7), good (0.7 to <0.8), very good (0.8 to <0.9), and excellent (0.9 to 1.0). In cases where multiple models were used in a study, the metrics for the best-performing model(s) were extracted. The collected data also included the strengths and limitations of the studies.

## 3. Results

**Figure 1:**
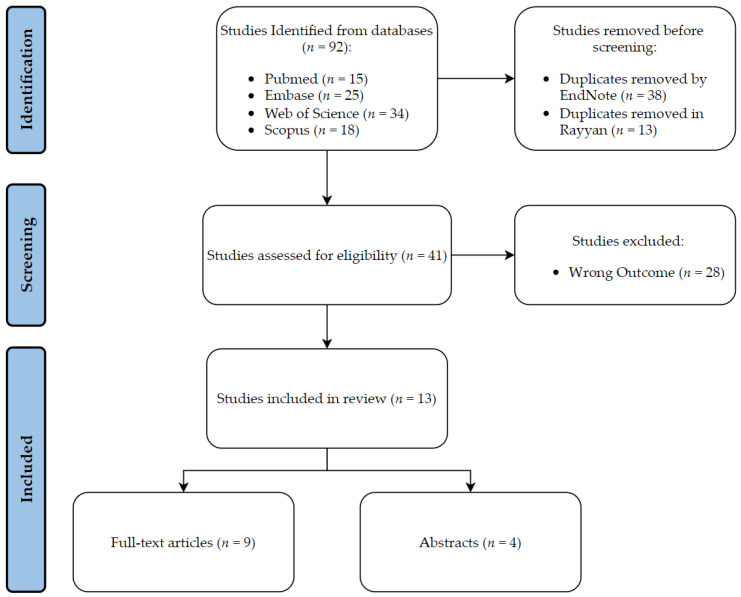
Schematic representation of the review process.

A total of 66 articles were identified through a search of the PubMed and EMBASE databases. Duplicate articles were removed using Endnote^®^ and Rayyan^®^ software, resulting in 43 articles, which were further screened using Rayyan^®^. After screening, 11 studies met the inclusion criteria and were categorized as diagnostic, prognostic, or treatment. Table 1 presents the outcome assessed, and the advantages and disadvantages of each study. The performance metrics for the best performing model in the included studies are summarized in Table 2. Details of the screening process are provided in Figure 1.

### 3.1. Diagnosis and Classification

Detecting CML and its stage is crucial for clinicians to avoid adverse consequences and to choose an optimal management plan for patients. In low- and middle-income countries (LMIC), clinicians mostly rely on microscopic manual inspection of bone marrow and blood smear films to diagnose and subtype leukemia. However, manual work is prone to error and takes time to formulate a diagnosis [11,34]. Therefore, multiple ML methods have been proposed to solve this issue by automating this step using an artificial intelligence-powered software to predict the diagnosis of CML.

Dese et al. developed an optical image processing software designed to automatically diagnose and subtype leukemia using the images of 250 stained blood smears. Images were optimized through preprocessing and the segmentation of white blood cells (WBC) from other structures. A total of 120 images with the diagnosis of CML were used in the study, of which 75 images were used for feature extraction and training, 30 for testing, and 15 for validation. ML algorithms were employed for feature extraction and identified several geometric (e.g., area, perimeter, aspect ratio), texture (e.g., contrast, correlation, homogeneity), color (mean hue saturation value), and statistical features (e.g., skewness, kurtosis, energy) that were accurate discriminators of leukemia subtype. Support vector machine (SVM), a supervised ML algorithm, was used as a classifier in the software. A confusion matrix was constructed to assess the performance on both the training and validation datasets. In both datasets, the software achieved an accuracy of 93.3% for classifying CML, and the diagnoses were provided in less than one minute. The system developed in this study shows the potential for replacing the manual methods of diagnosing leukemia using automated software. However, it has not been externally validated to evaluate its performance across different patient samples. Additionally, the study only reports the accuracy of detecting CML, and other important metrics such as sensitivity and specificity were not reported. These parameters would be useful to compare the performance of this software with other methods of CML classification such as manual classification and to consider its implementation in clinical practice [23].

Similarly, Cerrato developed an image processing algorithm to diagnose leukemia, which was trained on 1009 images of bone marrow aspirates and peripheral blood smears from patients diagnosed with leukemia through immunophenotyping. In a sample of 341 patients presenting symptoms of leukemia, the algorithm was applied and subsequently evaluated by an expert hematologist for external validation. The average time to obtain an initial diagnosis of leukemia was 75% within 24 h and 24% within 48 h. Out of the total sample, 20 patients (5.9%) received a preliminary diagnosis, of which four (20%) were diagnosed with CML. A 95% match was observed between the ML diagnosis and the immunophenotyping diagnosis. The use of this algorithm assisted hematologists in making early treatment decisions for patients, ultimately reducing the time needed to diagnose leukemia. This approach could increase the access to the diagnosis and treatment of leukemia, especially in lower income countries. Nevertheless, there is plenty of information missing from the abstract. For instance, there is no mention of the ML method used in developing the image processing software, or details on the training and testing phases of the ML model [24].

In 2016, Hempel and Fischer presented an interactive assistance system aimed to help clinicians in applying clinical practice guidelines (CPG) in the diagnosis and treatment of CML given their specific parameters (i.e., patient, equipment, medical experience). The proposed system was a self-learning server that stores information and learns to provide recommendations according to the most recent guidelines and expert opinions, which can actively encourage physicians to incorporate CPG into their daily practice. The system employs Bayesian inference and ML methods to stay up to date with the latest guidelines. No further data have been published about the outcomes of this system in the context of CML diagnosis and treatment [25].

**Figure 2:**
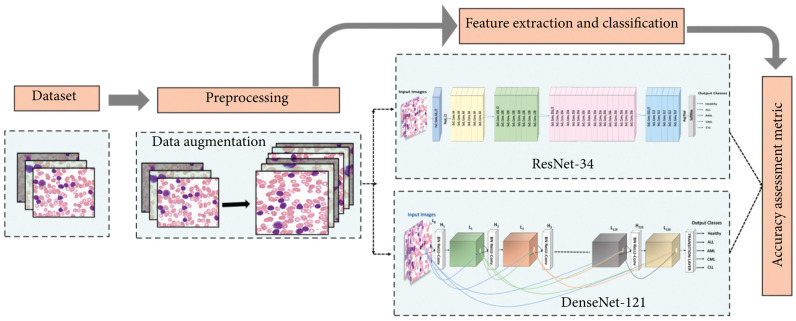
Proposed methodology for detecting and subtyping leukemia (Bibi et al., 2020) [26].

**Figure 3:**
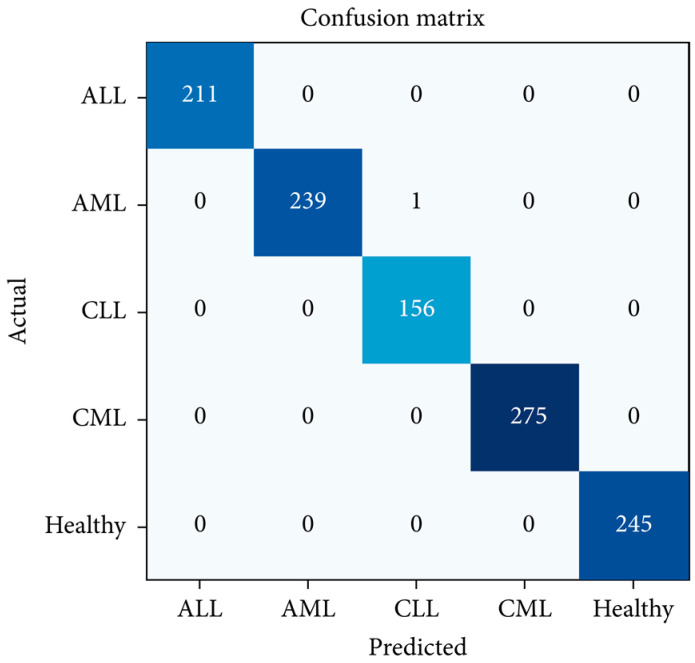
Confusion matrix of DenseNet-121 for leukemia subtype classification (Bibi et al., 2020) [26].

Bibi et al. created an Internet of Medical Things-based framework to detect and subtype leukemia from peripheral blood smear images using DenseNet-121 and ResNet-34 deep learning models (Figure 2). They trained the models on a dataset of 57 CML samples with 1243 images generated using various augmentation techniques. The CNN models directly extracted relevant features from the input images, eliminating the need for separate feature extraction. Despite unclear details on the training, testing, and validation datasets, the DenseNet-121 model had a perfect performance in predicting CML with 100% accuracy (Figure 3). The model achieved near-zero training and validation loss, demonstrating its ability to generalize to new data. The model outperformed previous models and has the potential for clinical implementation, but its efficiency and applicability require evaluation with additional patient samples including data on the time taken to classify images [26].

Haferlach et al. conducted a trial on 10,082 patients with suspected hematologic neoplasms. Skilled technicians independently labeled all peripheral blood smear samples, which were then reviewed by hematologists. Next, they trained a CNN model, an ImageNet-pretrained Xception model, on 8425 images to identify 21 predefined classes of neoplasms including CML. The model achieved 96% accuracy on the hold-out-set and 95% concordance with the pathogenic cases, as determined by manual inspection. Clinicians can utilize this cloud-based model for automated classification of scanned images of peripheral blood smears, thereby increasing the efficiency and reducing the cost of leukemia diagnosis [27].

**Figure 4:**
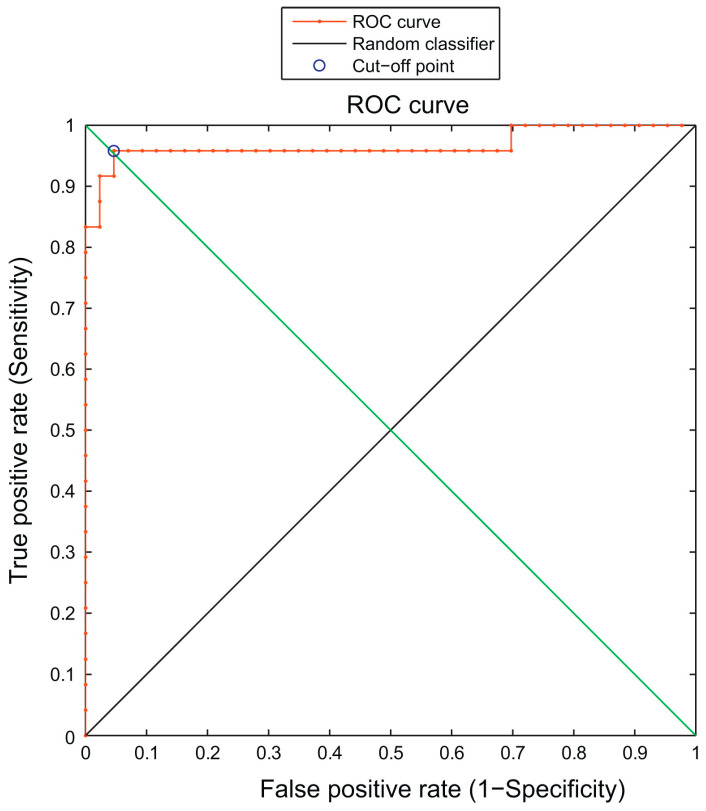
ROC curve analysis of the predicted probabilities of being normal mature neutrophils. The circle near the upper-left corner represents the cut-off point (51.79%) “Reproduced with permission from Ni et al., Computers in Biology and Medicine; published by Elsevier, 2013” [28].

Immunophenotyping using flow cytometry has been used in many hematological diseases as it is useful in quantifying and characterizing cells [35,36]. Unique antigen expressions can be used to classify cells into malignant or normal. This method is difficult to utilize in CML since mature neutrophils in CML patients can have a similar antigen expression behavior as the normal neutrophils [37,38]. Thus, polymerase chain reaction (PCR) is preferred to detect the presence of the BCR/ABL gene to diagnose CML. Ni et al. used SVM algorithms with flow cytometry data (four-color panel; CD45, CD65s, C15, CD11b) to analyze multiple cell parameters and classify them as mature malignant (CML) or normal neutrophils. Clinical diagnoses were confirmed through the PCR of BCR/ABL, immunophenotyping, and chromosome morphology. To train the model, data from nine CML patients and nine healthy donors were used, and data from 67 patients with various diagnoses were randomly assigned to the test group to assess the SVM model’s predictive ability. The model achieved a 95.5% overall accuracy, with a sensitivity and specificity of 95.8% and 95.3%, respectively, using a cut-off value of 51.79% predicted probability. The cut-off value was determined by the receiver operating characteristic (ROC) curve to have an AUC of 97% (Figure 4). This study utilized flow cytometry’s analytical ability to develop a model that accurately identifies mature neutrophil origin. However, larger and more diverse samples are needed to fully investigate the model’s usefulness in classifying neutrophil origin [28].

**Figure 5:**
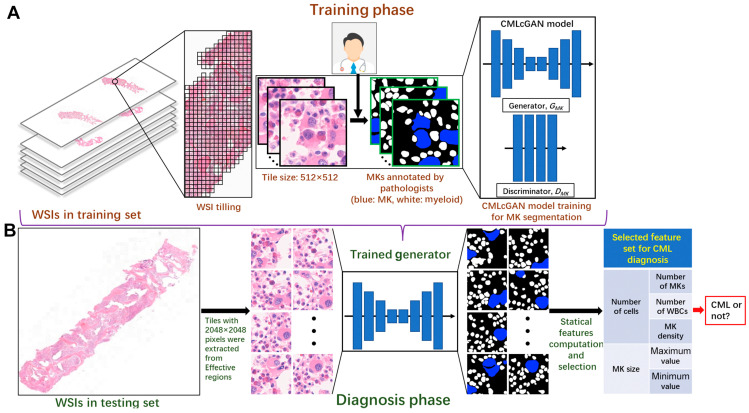
The framework of the automatic diagnosis of chronic myeloid leukemia (CML). (**A**) The CML conditional generative adversarial network (CMLcGAN) training. (**B**) The pipeline of the whole diagnostic process “Reproduced with permission from Zhang et al., The American Journal of Pathology; published by Elsevier, 2022” [29].

Zhang et al. used bone marrow specimens from 89 patients (58 CML patients, 31 controls) to developed a multiclass segmentation model to segregate the megakaryocytes (MKs) from myeloid cells using a conditional generative adversarial network (CMLcGAN). The model’s generator network utilized UNet++ and the discriminator network contained four 2× convolutional downsampling layers, and Figure 5 summarizes the framework used for the automatic detection of CML. Fivefold cross-validation was used to assess and compare the performance of the model. CMLcGAN was compared to seven models and outperformed with a mean dice coefficient of 81.8%, IoU of 71.2%, and pixel accuracy (PA) of 95.1%. Based on the segmentation results, seven characteristics (e.g., count, size, density) from MKs and the myeloid cells were analyzed using a *t*-test to determine the most important predictors for CML diagnosis. Five features were deemed important and were fed into eight different ML models. The linear SVM was shown to be the best-performing model to predict CML with an AUC of 84.93%. More studies with larger cohorts are needed to test the validity and feasibility of clinical implementation, as the study was conducted on a sample from a single diagnostic lab. Nevertheless, the study highlights the potential of using deep learning methods such as CMLcGAN for the precise segmentation and analysis of bone marrow cells, which could assist in diagnosing CML [29].

### 3.2. Prognosis

Despite the advancements in leukemia management, disease prognosis and survival prediction in CML patients can help in treatment decision-making and evading disease progression to advanced stages. It is known that the severity of blood cell abnormalities at the time of diagnosis is a prognostic factor in CML, and those in the early chronic phase of CML are more likely to maintain major molecular remission (MMR) after the discontinuation of tyrosine kinase inhibitors [34,39,40]. Therefore, an earlier diagnosis of CML could lead to a better prognosis.

A retrospective study conducted by Hauser et al. checked the predictive ability of the blood cell counts collected up to 5 years prior to diagnostic CML testing (BCR-ABL1 mutation test) in CML diagnosis. They utilized two ML models: extreme gradient boosting (XGBoost), which is based on decision trees, and least absolute shrinkage, and selection operator (LASSO), which is based on logistic regression. Data from 1623 patients were retrieved from the veterans’ health administration database to be incorporated into these models, then were split by a ratio of 80:20 into the training/validation and testing datasets, and the AUC and 95% confidence intervals were calculated for each model. The performance of the models trained on data gathered at the time of the BCR-ABL1 mutation was the most predictive (AUC 0.87–0.96), while that of the models trained on data gathered farthest from the mutation test was the poorest (2–5 years prior to the BCR-ABL1 test; AUC 0.59–0.67). This study provides evidence that the blood cell counts collected prior to BCR-ABL1 testing can predict future CML diagnosis, thus promoting earlier CML diagnosis and potentially improved prognosis. Although the study reports the AUC as a performance metric, providing additional metrics such as sensitivity and specificity would enhance the understanding of model performance and facilitate comparisons with other models. Furthermore, the data in this study were collected during routine medical care, which may have influenced the performance of the models. Therefore, future studies with a prospective study design and standardized methodology could improve the predictive accuracy of these models [30].

**Figure 6:**
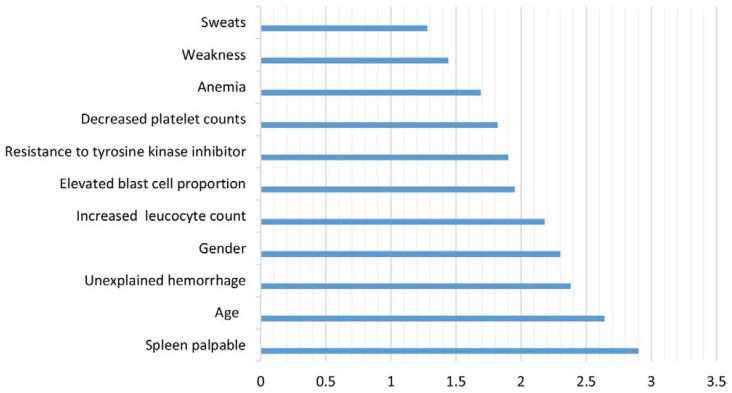
The most important variables selected by the mRMR (Shanbehzadeh et al., 2022) [31].

Shanbehzadeh et al. conducted a retrospective study to identify the most important factors affecting 5-year survival in CML and employed them in multiple ML algorithms to evaluate their ability to predict survival. Data on 45 variables associated with CML survival were extracted from an electronic medical record database for 837 patients, then randomly divided into training and testing groups in a ratio of 70:30. To maximize the efficiency and performance of predictive ML algorithms, the minimal redundancy maximal relevance (mRMR) feature selection algorithm was used to select the specified variables. Multiple ML methods were employed, extreme gradient boosting (XGBoost), k-nearest neighborhood (KNN), multilayer perceptron (MLP), J-48, pattern recognition network, probabilistic neural network, SVM (kernel = linear), and SVM (kernel = RBF). Twelve variables were selected to be fed into the ML algorithms (Figure 6), of which palpable spleen, age, and unexplained hemorrhage were the most relevant to CML 5-year survival. The SVM (kernel = RBF) outperformed in predicting the 5-year survival in CML among the eight models with 86% sensitivity, 85% specificity, 85.7% accuracy, and an AUC of 85%. This was a retrospective analysis of data from a single center, which may limit the applicability and generalizability of the models. Additionally, the retrospective nature of the study means that important variables that could improve the performance of ML models were not available. Further studies are necessary to enhance the quality and accuracy of these models [31].

One of the main goals of imatinib treatment in patients with chronic phase CML (CP-CML) is achieving MMR within two years of the diagnosis because it is associated with improved prognosis and survival [41,42,43,44]. Studies have shown that the EUTOS long-term survival (ELTS) score is a good predictor of survival in CML patients [45,46]. A model built by Kok et al. utilized multiple bioassays to classify CML-CP patients into high-risk (HR) and low-risk (LR) groups and to compare it to the ELTS score to determine which is a better predictor of MMR. In addition, they combined the bioassay model with the ELTS score to check if it provided an added predictive value. The model used data from 210 TIDEL-II (frontline imatinib with early switch to nilotinib for failure to meet the optimal time-dependent molecular targets) patients, of which 201 patients had ELTS scores. Out of all the bioassays included in the study, the recursive partitioning and regression trees (rpart) algorithm identified only four to be the most important predictors of MMR (IGFBP2 gene expression, KIR2DL5B genotype, OA, and MCP-1 cytokine plasma level). The model achieved an accuracy of 84% with those classified as high risk having significantly lower rates of MMR than those classified as low risk (26% vs. 86%, *p* < 0.0001) as well as higher for blast-crisis progression (15% vs. 1.6%, *p* = 0.006). This model can be used along with the ELTS score to risk-stratify CML-CP patients receiving TIDEL-II treatment, but it still requires external validation with larger samples to assess performance in the clinical context [32].

### 3.3. Treatment

Tyrosine kinase inhibitors (TKIs) improved the survival of patients with CML-CP [47,48]. Many patients have to change into another TKI throughout the course of therapy. The decision of which TKI is best for a patient is based on multiple biological and sociodemographic features [41,49]. Sasaki et al. created the LEukemia Artificial Intelligence Program (LEAP) to provide treatment recommendations for CML-CP patients based on the XGBoost ML algorithm. They included a cohort of 630 patients who received TKIs from a single center and were randomly assigned into training (*n* = 504) and test cohorts (*n* = 126) in a 4:1 ratio. Data on 101 variables from the training cohort were utilized in a 3-fold cross-validation step to develop the LEAP ML model. After hyperparameter tuning, the LEAP model achieved an AUC of 0.82 in the test cohort. To test the model’s validity, the test group was further divided into those who received LEAP-recommended TKIs (*n* = 94 (75%)) and those who did not (*n* = 32 (25%)). The median follow-up period for the total cohort in the study was 139 months. Complete cytogenetic response (CCyR) was achieved in 89% of the LEAP-recommended group compared with 81% in the other group. Moreover, MMR was achieved in 82% and 75% in the LEAP-recommended group and LEAP non-recommended group, with overall survival rates of 98% and 77% (*p* < 0.001), respectively. While the study has the potential to improve patient outcomes, it has limitations. For example, it did not include data on bosutinib, and the LEAP model could only make recommendations for first-line treatment. Further data are needed to develop a model for recommending treatment after the first-line has failed [33,50,51].

## 4. Discussion

CML is a major component of leukemias, accounting for about 15% of all leukemia cases. Many aspects of CML (e.g., workup, management, risk assessment) can be improved, especially in low-income countries with poor access to advanced technologies and expensive treatments. Multiple ML models have been proposed to better these aspects and to make the diagnosis and management of CML more accessible and affordable. This review aimed to highlight the different implementations of ML algorithms in diagnosis prediction, risk assessment, and the management of CML.

This review has summarized the findings of 11 studies pertaining to CML diagnosis, prognosis, and treatment using different ML algorithms to automate these processes. Most of the studies included in this review were concerned with ML models predicting the diagnosis of CML based on images of peripheral blood smears or bone marrow aspirates. Moreover, one study utilized flow cytometry to subclassify neutrophils as CML mature neutrophils or normal mature neutrophils. The ML algorithm used in this study achieved high accuracy in predicting the origin of neutrophils. These were followed by studies addressing the prognosis of CML by employing ML to stratify patients with CML according to risk or to predict their survival. Finally, a study demonstrating a model that can provide individualized treatment recommendations for patients with CML-CP.

Most studies in this review achieved a high performance after cross-validation using the test/validation groups. However, this only represents the internal validation as the test/validation groups were usually derived from the same population that the training groups were derived from. This means that these results still await external validation using samples from populations with different characteristics than the original studies. Moreover, some studies had small sample sizes with data derived from a single center/lab or data collected in a different manner than that of the clinical practice. All these can pose a threat to the applicability and generalizability of the results of these studies. Therefore, studies examining these models on bigger and more diverse samples are needed to make sure these models can be applied in different clinical settings.

## 5. Conclusions

The literature contains several studies utilizing ML algorithms in CML to improve the diagnosis, prognosis, and management. Many of the models in these studies achieved a high precision in performing their tasks. However, further research with bigger samples and better methodology is still needed; this can be achieved by implementing these models in various clinical contexts to test their performance. Finally, it is important to note that the implementation of ML models in this context has the potential of significantly helping physicians by providing early diagnosis, better risk assessment, and individualized treatment plans, which will positively affect the care of patients with CML.

## Figures and Tables

**Table 1:** Data extraction summary of the included studies.

Study	Outcome	Advantages	Disadvantages
Dese et al. (2021) [23]	Diagnosing and subtyping leukemia	- High performance (93.3% ACC) - Rapid (<1 min) - Lower cost than manual diagnosis - ML algorithms used in feature extraction to optimize performance of the classifier (i.e., SVM)	- No mention of thresholds used in the confusion matrix - No data on sensitivity or specificity in detecting CML - No external validation of the software
Cerrato (2020) [24]	Diagnosing and subtyping leukemia	- Externally validated - High performance (95% concordance) - Faster diagnosis than traditional methods in low-income countries - Can use blood smears or bone marrow aspirates	- Absent data on the methodology of developing the software - Only concordance with immunophenotyping was reported as a performance metric
Hempel and Fischer (2016) [25]	Applying clinical practice guidelines	- Easy access from Internet browsers - Combines guidelines and expert opinions - Self-learning through Bayesian inference or ML algorithms	- No follow-up data on the project were published - No discussion of the ML methods used in developing the software - No discussion of the system’s performance assessment methodology
Bibi et al. (2020) [26]	Diagnosing and subtyping leukemia	- Use of CNN deep learning models - Best performing model in detecting and subtyping leukemia (100% ACC) - Near-zero training and validation loss	- No data on time taken to reach diagnosis - No clear description of training, testing, validation datasets
Haferlach et al. (2021) [27]	Diagnosing and subtyping hematological malignancies	- Easy access - High performance (96% ACC against hold-out-set) - Can detect several malignancies other than leukemia - Large sample size of diverse hematological diseases	- External validation of the results is needed - No data reported on the sensitivity or specificity of the model - Hold-out was used instead of cross-validation
Ni et al. (2013) [28]	Distinguishing malignant CML neutrophils vs. normal neutrophils	- High performance (95.8% SEN, 95.3% SPE, 95.5% ACC) - Externally validated - Utilizes flow cytometry	- Further testing on diverse patient samples is needed
Zhang (2022) [29]	Segmentation of bone marrow cells	- High performance (81.8% dice coefficient, 71.2% IoU, 95.1% PA) - Compares multiple models in segmentation performance - Clinical cross-validation using eight ML models	- Studies with larger, more diverse samples are needed
Hauser (2021) [30]	Predicting CML diagnosis	- High performance (average AUC 0.63–0.92) - Uses blood counts - Can promote better prognosis through early detection	- Retrospective analysis - Studies with standardized data collection methods are needed - Only AUC was reported as performance metric
Shanbehzadeh et al. (2022) [31]	Predicting 5-year CML survival	- Comparison of multiple ML models - Moderate performance (86% SEN, 85% SPE, 85.7% ACC, 85% AUC)	- Retrospective analysis - Single center study - Prospective studies are needed to improve the model’s performance
Kok et al. (2018) [32]	Risk-stratification of CML-CP patients	- Moderate accuracy in predicting MMR (84%) - Combines risk scores with bioassays - Adds to the predictive value of ELTS score	- Studies with larger samples are required to validate the results
Sasaki et al. (2021) [33]	Making treatment recommendations for CML-CP patients	- Higher survival in treatment-recommended group (98% vs. 77%) - Potential to improve patient outcomes - Recommendations individualized to each patient	- Single center study - Study did not include data on bosutinib - Further studies are needed to expand the model’s recommendations after failure of first-line treatment

ML, machine learning; SVM, support vector machines; CNN, convolutional neural networks; CML, chronic myeloid leukemia; CML-CP, chronic myeloid leukemia chronic phase; MMR, major molecular remission; ELTS, EUTOS long-term survival; IoU, intersection-over-union; PA, pixel accuracy; ACC, accuracy; SEN, sensitivity; SPE, specificity; AUC, area under the curve.

**Table 2:** Performance metrics for the best models in the included studies.

Study (Year)	Outcomes	Best Models	AUC	ACC	SEN	SPE	Concordance
Dese et al. (2021) [23]	Diagnosing and subtyping leukemia	SVM	NR	93.33%	NR	NR	NR
Cerrato (2020) [24]	Diagnosing and subtyping leukemia	NR	NR	NR	NR	NR	95%
Hempel and Fischer (2016) [25]	Applying clinical practice guidelines	NR	NR	NR	NR	NR	NR
Bibi et al. (2020) [26]	Diagnosing and subtyping leukemia	ResNet-34	NR	99.73%	NR	NR	NR
		DenseNet-121	NR	100%	NR	NR	NR
Haferlach et al. (2021) [27]	Diagnosing and subtyping hematological malignancies	Xception	NR	96%	NR	NR	95%
Ni et al. (2013) [28]	Distinguishing malignant CML neutrophils vs. normal neutrophils	SVM	97%	95.5%	95.8%	95.3%	NR
Zhang (2022) [29]	Segmentation of bone marrow cells	cGAN	NR	95.1% *	NR	NR	NR
		Linear SVM	84.93%	NR	NR	NR	NR
Hauser (2021) [30]	Predicting CML diagnosis	XGBoost	87–95% †	NR	NR	NR	NR
		LASSO	91–96% †	NR	NR	NR	NR
Shanbehzadeh et al. (2022) [31]	Predicting 5-year CML survival	SVM (kernel = RBF)	85%	85.7%	86%	85%	NR
Kok et al. (2018) [32]	Risk-stratification of CML-CP patients	rpart	NR	84%	NR	NR	NR
Sasaki et al. (2021) [33]	Making treatment recommendations for CML-CP patients	XGBoost	82%	NR	NR	NR	NR

SVM, support vector machine; ResNet, Residual networks; DenseNet, Densely connected convolutional network; Xcpetion, Extreme inception; cGAN, conditional Generative adversarial network; XGBoost, extreme gradient boosting; LASSO, least absolute shrinkage and selection operator; rpart, Recursive partitioning and regression trees; AUC, area under ROC curve; ACC, accuracy; SEN, sensitivity; SPE, specificity; NR, not reported; *, Pixel accuracy; †, At time of diagnosis.

## Data Availability

Not applicable.
